# 铁卟啉金属有机框架材料用于高效富集地龙多肽

**DOI:** 10.3724/SP.J.1123.2025.11018

**Published:** 2026-08-08

**Authors:** Kunsong ZHAN, Mingzhu TANG, Ying LIN, Linjun NI, Lishuang YU

**Affiliations:** 1. 福建中医药大学药学院，福建 福州 350122; 1. College of Pharmacy，Fujian University of Traditional Chinese Medicine，Fuzhou 350122，China; 2. 苏州大学药学院，江苏 苏州 215000; 2. College of Pharmaceutical Science，Soochow University，Suzhou 215000，China

**Keywords:** 地龙, 多肽, 分离纯化, 金属有机框架材料, 高效液相色谱, earthworm, polypeptides, separation and purification, metal-organic frameworks （MOFs）, high performance liquid chromatography （HPLC）

## Abstract

地龙为我国常用的动物类中药，蛋白多肽类是其主要的活性成分，但现有分离检测方法存在制备能力不足、分离重现性较差等问题。本研究采用铁卟啉金属有机框架材料PCN-222（Fe）特异吸附地龙多肽成分L-PeakⅡ，吸附容量为139 mg/g，然后进一步将PCN-222（Fe）作为固相吸附剂，并结合液相色谱技术从复杂中药材地龙酶解液中快速分离并制备多肽成分L-PeakⅡ。为了获得地龙多肽富集的最佳实验条件，对酶解液质量浓度、富集过程pH值、洗脱溶剂类别、洗脱时间等影响因素进行了研究和优化。结果表明，当酶解液质量浓度为3.0 mg/mL、富集过程pH值为9、洗脱溶剂为含0.2%三氟乙酸的50%乙腈水溶液、洗脱时间为2 h时，富集效果最佳。最后通过高压制备色谱仪制备得到纯度达到95%以上的地龙多肽组分。本文所构建的金属有机框架材料PCN-222（Fe）联用色谱法可以实现地龙药材样品中多肽成分L-PeakⅡ的高效制备，可为复杂动物药多肽成分的分离纯化提供参考。

## Abstract (en)

Earthworms are a commonly used animal-derived traditional Chinese medicine in China， with proteins and polypeptides as their main active ingredients. However， the existing methods for separating and detecting earthworm polypeptides have problems such as insufficient preparation capacity and poor separation reproducibility. To address these challenges， this study used the iron porphyrin metal-organic framework material PCN-222（Fe） to specifically adsorb the earthworm polypeptide component L-PeakⅡ， with an adsorption capacity of 139 mg/g. First of all， PCN-222（Fe） material was successfully prepared via the one-pot method. The earthworm protease hydrolysate was obtained through alkali extraction and acid precipitation， and trypsin enzymatic hydrolysis. Then， PCN-222（Fe） was further used as a solid-phase adsorbent and combined with liquid chromatography to rapidly separate and prepare the polypeptide component L-PeakⅡ from complex earthworm enzymatic hydrolysate of the Chinese medicine. Qualitative analysis was performed by HPLC， and quantitative analysis was conducted with a BCA assay kit. HPLC analysis of the earthworm protease hydrolysate revealed multiple chromatographic peaks. After enrichment with PCN‑222（Fe） and subsequent elution， the eluted sample exhibited fewer peaks， with a well‑defined peak appearing at a retention time of approximately 15 min. In order to obtain the optimal experimental conditions for the enrichment of earthworm polypeptides， influencing factors including enzyme hydrolysate mass concentration， pH value， elution solvent type， and elution time were investigated and optimized. The results showed that optimal enrichment was achieved by incubating at an enzymatic hydrolysate mass concentration of 3.0 mg/mL and pH of 9， followed by elution with 50% acetonitrile containing 0.2% trifluoroacetic acid for 2 h. Finally， the earthworm-derived polypeptide component was obtained using a high-pressure preparative chromatographic system， achieving a purity exceeding 95%. The integrated approach employed in this study， which combines PCN-222（Fe） material with preparative chromatography， enables the efficient preparation of the target polypeptide L-PeakⅡ from medicinal earthworm samples. This strategy provides a valuable reference for the separation and purification of polypeptide components from complex animal-derived traditional Chinese medicines. Moreover， it offers a more robust， scalable， and reproducible pathway for harnessing the therapeutic potential of natural product peptides， thereby bridging the gap between traditional medicine and modern pharmaceutical analysis.

目前，蛋白多肽类物质的分离与纯化方法主要包括色谱法（如反相高效液相色谱（RP-HPLC）、亲和色谱、离子交换色谱等）^［1］^、膜分离法^［2］^、离心过滤法等。而近年来，有机框架材料因其在多肽类生物分子的选择性富集方面展现出显著优势，逐渐成为生物分析与药物分离研究中的热点。该类材料主要包括金属有机框架材料（metal-organic frameworks，MOFs）^［3］^、共价有机框架材料（covalent organic frameworks，COFs）^［4］^以及氢键有机框架材料（hydrogen-bonded organic frameworks，HOFs）^［5］^。其中MOFs是由金属簇与有机配体通过配位作用构建而成的，具有高度有序的多孔结构。这类材料在水、热、酸碱及有机溶剂中均表现出卓越的稳定性，在药物分离与分析领域具有广阔的应用前景^［6］^。例如Mensinger等^［7］^利用MOFs材料HKUST-1和MIL-69吸附分离淀粉样蛋白*β*肽（A*β*），该材料通过暂时隔离多肽然后再释放，能够在1 h内迅速吸附A*β*并实现快速分离。以铁修饰的PCN-222（Fe） MOFs材料可构筑出具有多级孔道结构的三维骨架，展现出较大的比表面积及良好的分子可达性。其在pH 3~10范围内稳定，具备优异的热力学、力学及化学稳定性，因而在多肽类生物活性分子的选择性吸附与富集方面具有显著优势^［8］^，但目前暂无关于PCN-222（Fe）应用于多肽富集的报道。

地龙为我国传统中药材，可用于治疗高热神昏等病症^［9］^。其富含人体所需营养物质，包括蛋白质、核苷酸、微量元素、维生素等，其中蛋白质是地龙的主要成分，占比约为其干重的56%～66%^［10］^。与一般植物蛋白相比，地龙蛋白、多肽的氨基酸模式更接近人类蛋白，更容易被人体利用^［11］^。此外，现代药理学研究证实，地龙蛋白多肽类物质具有抗血栓^［12］^、抗氧化^［13］^、抗纤维化^［14］^、抗疲劳^［15］^、促进创面愈合^［16］^等丰富的药理作用，针对其成分进行研究探索具有重要意义。目前报道的地龙多肽分离检测方法主要是毛细管电泳法和高效液相色谱法等^［17-19］^。例如颜松益等^［18］^建立了一种简单灵敏的毛细管电泳-大体积样品堆积法同时分离检测4种地龙多肽，但其存在制备能力差、分离重现性不好等缺点。Li等^［19］^采用反相高效液相色谱法，从蚯蚓的皮肤分泌物中分离出一种新的类腰痛素抗菌肽，但存在样品处理过程繁琐等问题。

本文以地龙为研究对象，探讨PCN-222（Fe）对地龙酶解液中多肽的富集能力。通过合成的PCN-222（Fe）材料特异性吸附地龙多肽，并结合色谱技术从地龙酶解液中快速分离并制备得到一个地龙多肽成分（命名为L-PeakⅡ），并系统优化了酶解液质量浓度、pH值、洗脱溶剂类型及洗脱时间等关键参数，建立了一种基于PCN-222（Fe）结合RP-HPLC技术的地龙多肽分离新策略。该方法旨在开发一种简便、高效且具有良好选择性的地龙多肽分离富集体系，为天然药物活性成分的分离与分析提供新的技术手段与方法学支持。

## 1 实验部分

### 1.1 仪器与试剂

EazySep^®^-3030高效液相色谱仪（上海通微分析技术有限公司）；AUW120D型电子天平（日本岛津公司）；DZF-6050真空干燥箱（上海精宏实验设备有限公司）；XH-C旋涡混合器（金坛市白塔新宝仪器厂）；CV600冷冻离心浓缩仪（北京吉艾姆科技有限公司）；TGL-16G高速离心机（上海安亭科学仪器厂）；LC-8A高压制备色谱仪（日本岛津公司）；循环水式多用真空泵（郑州长城科工贸有限公司）；KQ-500DE数控超声波清洗器（昆山市超声仪器有限公司）；SU-20 UV-实验室纯水系统（上海骇思仪器科技有限公司）；HSC-12B氮吹仪（天津恒奥科技有限公司）；PHS-3C pH计（上海仪电科学仪器股份有限公司）；SU-8010扫描电子显微镜（SEM，日本理学株式会社）；Minifelx X-射线衍射仪（XRD，日本理学株式会社）；Nicolet iS20傅里叶红外光谱仪（FT-IR，美国赛默飞世尔科技公司）**。**

氯化锆（ZrCl_4_）购于上海麦克林生化科技股份有限公司；氢氧化钠（NaOH）、碳酸氢铵（NH_4_HCO_3_）、*N*，*N*-二甲基甲酰胺（DMF）、甲醇、丙酮、无水乙醇均为分析纯，购于国药试剂有限公司；铁（Ⅲ）-四（4-羧基苯基）氯化卟啉（TCPP-Fe（Ⅲ））、*N*，*N*-二乙基甲酰胺（DEF）购于上海泰坦科技股份有限公司；乙腈（ACN）、三氟乙酸（TFA）为色谱纯，购于天津市康科德科技有限公司；BCA蛋白定量试剂盒购于北京兰博利德商贸有限公司；乙酸（AcOH）、甲酸（HCOOH）为分析纯，购于西陇科学股份有限公司；胰蛋白酶购于上海麦克林生化科技股份有限公司（来自牛胰脏，效力≥2 500 units/mg）；实验用水为超纯水；地龙中药饮片，购于亳州市谯郡堂中药饮片有限公司**。**

### 1.2 PCN-222（Fe）材料合成

参考文献方法合成PCN-222（Fe）^ ［8］^，具体操作如下：准确称取300 mg ZrCl_4_和200 mg TCPP-Fe（Ⅲ）于烧杯中，并加入32 mL DEF，超声溶解5 min。加入10.8 g苯甲酸，超声溶解10 min，转移至100 mL反应釜中，烘箱120 ℃反应48 h。室温冷却后，转移至离心管，以8 000 r/min转速离心10 min，弃去上清，收集沉淀。

沉淀部分进行洗涤和活化处理，具体操作如下：将沉淀转移至烧杯中，加入10 mL DMF，混匀，烘箱100 ℃反应2 h，室温冷却，8 000 r/min离心10 min，分离沉淀。再加入10 mL DMF，涡旋分散，离心弃去溶剂，重复操作2次。所得沉淀再加入2 mL丙酮，40 ℃烘箱放置2 h，转移至离心管，8 000 r/min离心10 min，分离沉淀。加入2 mL丙酮，涡旋分散，离心弃去溶剂，重复操作2次。收集沉淀，室温干燥，即得暗紫色粉末状的PCN-222（Fe）材料，室温保存，用于表征和后续富集实验。

### 1.3 地龙蛋白酶解液制备

干燥地龙药材经粉碎机粉碎，称取药材粉末10 g，加入100 mL超纯水，搅拌均匀，用1 mol/L氢氧化钠溶液调节上述溶液的pH值为10，超声（250 W）提取1 h。用纱布过滤两次，收集滤液，用1 mol/L盐酸溶液调节pH值为4，转移至4 ℃冰箱中静置2 h，使地龙蛋白沉淀。再转移至离心管以4 000 r/min转速离心10 min，收集沉淀，用超纯水洗涤两次。40 ℃干燥箱减压干燥10 h，得地龙粗蛋白。

精确称量地龙粗蛋白50 mg，加入10 mL碳酸氢铵溶液（25 mmol/L），超声10 min使蛋白分散。然后加入25 mg胰蛋白酶，摇匀，于37 ℃恒温水浴锅酶解20 h。待酶解结束后，90 ℃水浴处理10 min，使胰蛋白酶灭活。放冷后3 000 r/min离心5 min，收集上清液，转移至冷冻离心浓缩仪中冷冻干燥24 h，即得地龙蛋白酶解液冻干粉末。使用时用超纯水复溶，氢氧化钠/盐酸溶液调节pH，可得到所需质量浓度和pH值的地龙蛋白酶解液（enzymatic hydrolysate）。

### 1.4 PCN-222（Fe）材料富集地龙多肽

精密称取PCN-222（Fe）材料3 mg，加入蛋白酶解液3 mL（质量浓度为3.0 mg/mL，pH=9），涡旋混匀，于摇床上振摇10 min进行富集。然后10 000 r/min离心10 min，分离上清，即为富集上清液。沉淀部分加入1 mL含0.2% TFA的50%乙腈水溶液，涡旋混匀，于摇床上振摇2 h进行洗脱，洗脱结束后10 000 r/min离心10 min，分离上清，即为富集洗脱液（eluent）。

### 1.5 吸附容量的测定

通过BCA蛋白定量试剂盒测定样品中的肽含量，并计算PCN-222（Fe）材料对于地龙多肽的吸附容量。具体操作如下：首先，根据试剂盒说明书，将试剂A与试剂B按50∶1体积比混合，涡旋混匀，配制得到反应工作液。使用PBS溶液稀释BSA蛋白标准品溶液，得到不同质量浓度的标准品溶液（0、25、125、250、500、750、1 000、1 500、2 000 μg/mL）。然后各待测样品和标准品溶液各取20 μL至96孔板中，再加入200 μL/孔反应工作液，混匀，37 ℃反应30 min。使用酶标仪测定562 nm处的吸光度值（*Y*），以吸光度值为纵坐标，标准品质量浓度（*X*）为横坐标，拟合得线性方程，将待测样品吸光度值代入方程计算得样品多肽质量浓度。根据公式（1）计算材料对于地龙蛋白多肽类物质的吸附容量（*Q*_e_，mg/g）


$$Q_{e}=\frac{\left(C_{0}-C_{e}\right)\times V}{m}$$ （1）

式中：*C*_0_为酶解液总肽质量浓度（mg/mL）；*C*_e_为富集上清液总肽质量浓度（mg/mL）；*V*为进行富集的酶解液体积（mL）；*m*为PCN-222（Fe）材料的质量（g）。

### 1.6 HPLC分析

使用液相色谱分析酶解液和富集洗脱液，评估PCN-222（Fe）材料的富集效果，分析条件如下：色谱柱为Agilent ZORBAX 300SB-C18柱（250 mm×4.6 mm，5 μm），流动相A为乙腈，流动相B为0.1% TFA水溶液，柱温30 ℃，检测波长220 nm，进样量20 µL，流速1.0 mL/min，梯度洗脱程序为0~30 min，95%B→75%B；30~32 min，75%B→95%B；32~42 min，95%B。

### 1.7 地龙多肽纯化组分的制备

将洗脱样品氮吹浓缩后，于LC-8A高压制备色谱仪上进行制备，制备条件如下：色谱柱为中谱红RD-C18柱（250 mm×10.0 mm，5 µm），流动相A为乙腈，流动相B为0.1%TFA水溶液，以80%B条件等度洗脱，流速3 mL/min，柱温30 ℃，检测波长220 nm，进样量500 µL。制备得到的纯化组分用1.6节HPLC条件进行分析。

## 2 结果与讨论

### 2.1 PCN-222（Fe）材料的表征

使用扫描电子显微镜、X射线衍射仪和傅里叶红外光谱仪对合成材料进行表征（图1）。如图1a所示，所合成材料为棒状晶体形态，边缘大致为六边形，和PCN-222（Fe）的结构空间群（P6/mmm）相符^［20，21］^。材料XRD图谱（图1b）与其他文献中报道的相似^［22，23］^，在2*θ*=6.6°、7.1°、8.2°、9.6°、9.8°处具有特征衍射峰，与剑桥晶体数据中心（CCDC）收载的PCN-222（Fe）（ID：893545）的图谱数据相吻合。图1c为合成材料的红外光谱图。研究所合成材料主要衍射峰与前期报道较相符，在1 176、1 600 cm^‒1^处的吸收峰显示为材料卟啉配体大环骨架特征吸收，1 654、1 412 cm^‒1^处为羧基-COO-变形振动峰，719、869 cm^‒1^处显示为苯环C-H键平面外弯曲振动信号峰^［22-24］^。表征结果证明PCN-222（Fe）材料的成功合成。

**图1:**
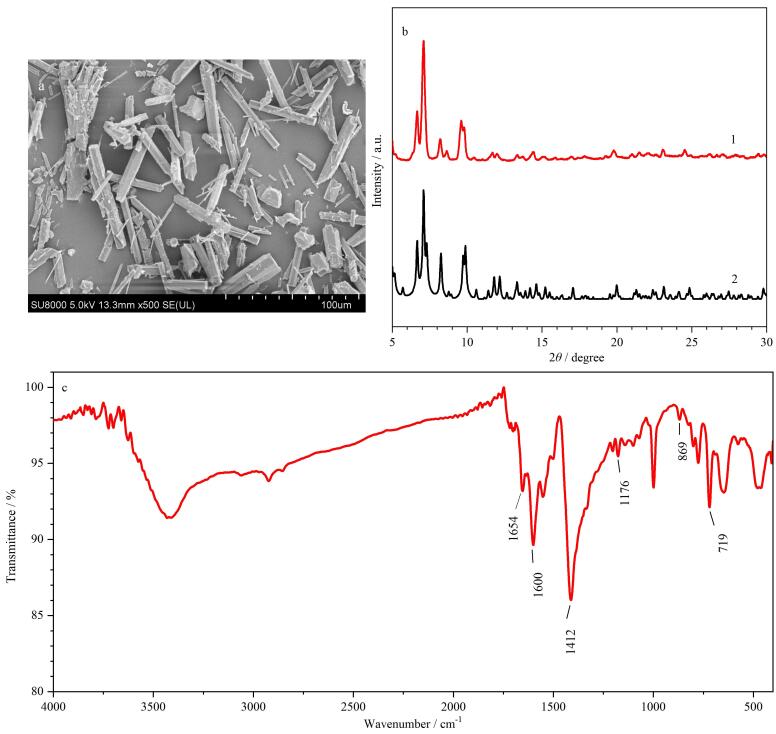
PCN-222（Fe）的（a）SEM图、（b）XRD图以及（c）FT-IR光谱图

### 2.2 地龙多肽的提取与富集

蛋白酶酶解法是提取活性肽最常用的方法，即通过一种或者多种蛋白酶将蛋白质酶解为小片段的氨基酸序列，该方法条件温和、可重复性强。本研究通过碱提取酸沉淀法获得地龙蛋白，再通过胰蛋白酶进行酶解以获得肽段。图2中地龙蛋白酶解液的色谱图含有多个色谱峰，说明经过胰酶酶解后，得到的地龙多肽片段较丰富。

**图2:**
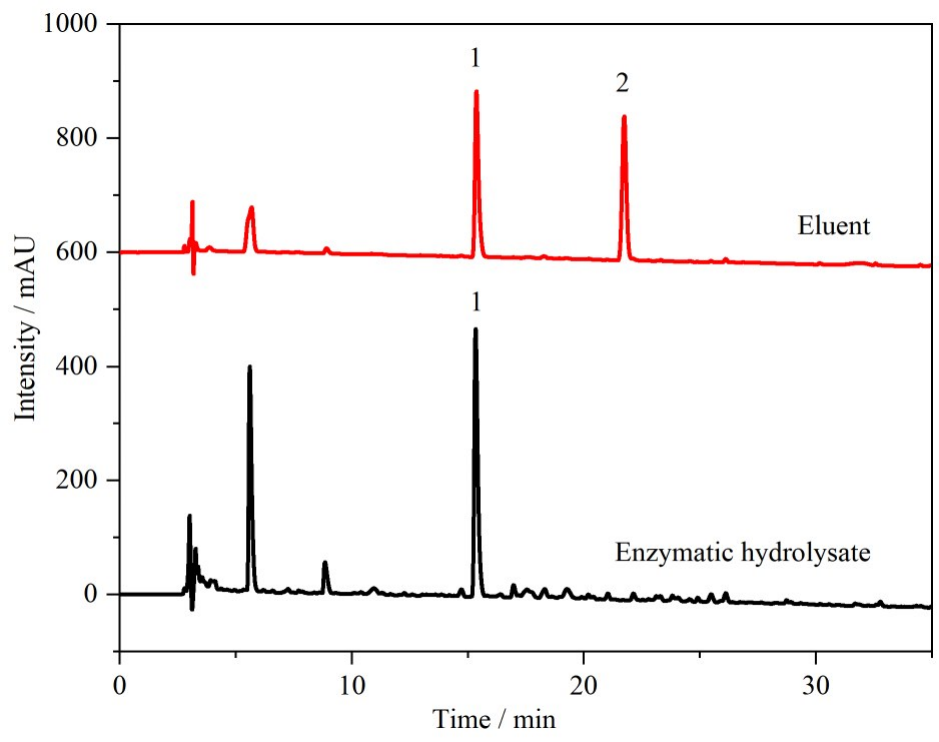
材料富集洗脱液与地龙蛋白酶解液的色谱图

从复杂的酶解液中分离纯化得到纯度更高的肽组分，是理化性质分析、活性研究以及结构解析等工作的基础。但传统的分离方法具有操作复杂、分析周期长等问题。近些年，随着新材料的研发与应用，越来越多的材料在成分分离领域得到了应用。本研究合成了锆基MOF材料PCN-222（Fe），并将其用于富集酶解液中的地龙肽。如图2所示，和酶解液的色谱图相比，经过PCN-222（Fe）富集和洗脱后得到的洗脱样品，色谱峰数量明显减少，表明材料处理可以实现复杂酶解液的纯化。洗脱样品在15.37 min位置具有和酶解液保留时间（15.34 min）一致的色谱峰，说明材料对该成分具有富集效果，将该色谱峰成分命名为L-PeakⅡ。通过材料空白对照的液相色谱图，表明洗脱样品中色谱峰2来自PCN-222（Fe）材料。通过BCA蛋白定量试剂盒对样品的肽浓度进行定量，测得地龙蛋白酶解液中总肽质量浓度为1.113 mg/mL，富集上清液中总肽质量浓度为0.974 mg/mL，富集洗脱液中总肽质量浓度为0.583 mg/mL。通过公式（1）计算得PCN-222（Fe）对地龙肽的吸附容量为139 mg/g。BCA蛋白定量试剂盒测定结果为阳性，可说明样品中含有蛋白多肽成分。结果表明，PCN-222（Fe）能实现从复杂的酶解样品中快速分离多肽，较传统方法高效简便。

### 2.3 富集条件的优化

为了获得更优的富集效果，通过单因素实验考察了酶解液质量浓度、体系pH、洗脱溶剂及洗脱时间对多肽L-PeakⅡ富集效果的影响。

#### 2.3.1 酶解液质量浓度优化

实验考察了3 mg PCN-222（Fe）对于3 mL不同质量浓度（0.5、1.0、2.0、3.0、4.0 mg/mL）酶解液的富集效果。结果如图3a所示，随着酶解液质量浓度的增加，多肽L-PeakⅡ的峰面积逐渐增大，当质量浓度达到3.0 mg/mL时，峰面积趋于平衡，表明材料对于L-PeakⅡ的吸附达到饱和。因此综合考虑，选择3.0 mg/mL作为最适酶解液质量浓度。

**图3:**
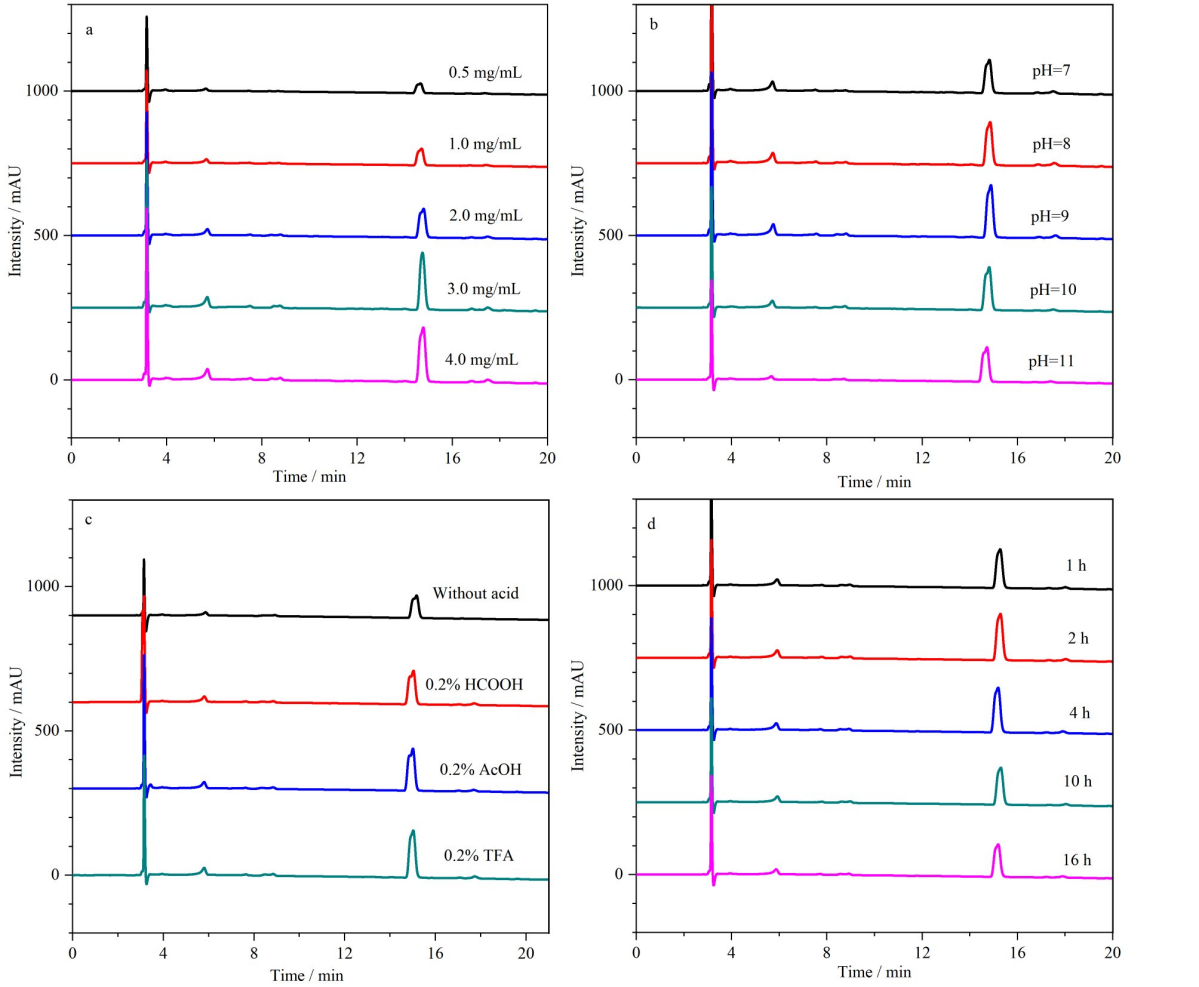
不同（a）酶解液质量浓度、（b）酶解液pH值、（c）洗脱溶剂类型以及（d）洗脱时间条件下富集洗脱液的色谱图

#### 2.3.2 富集过程pH值优化

pH值影响多肽在溶液中的稳定性，并且会对PCN-222（Fe）材料的晶体结构产生影响，据报道^［22］^，在pH=6.4时观察到PCN-222（Fe）的电荷为0，在该值以外的pH值下，材料表面带有电荷。而多肽与蛋白质在理化性质上具有相似性，在不同pH环境下分别以不同带电形式存在，这种电荷变化对多肽的稳定性和功能都有重要影响。因此富集过程pH值也是影响富集效果的重要因素。考察富集过程中不同pH值（7、8、9、10、11）对富集效果的影响，如图3b所示，富集过程中pH值在7~9时，多肽L-PeakⅡ的峰面积随着pH值的增大而增大，当pH值大于9时（pH 10、11）多肽L-PeakⅡ的峰面积开始下降。结果表明pH为9时，PCN-222（Fe）对于L-PeakⅡ成分具有最佳吸附效果，故选择富集过程中溶液环境pH值为9作为最佳条件。

#### 2.3.3 洗脱溶剂类别优化

材料富集过程中会受到多种因素的影响，而洗脱液中L-PeakⅡ的含量很大程度上取决于洗脱溶剂的类别。前期预实验表明，50% ACN水溶液对L-PeakⅡ具有较好的洗脱效果，基于此，研究比较了HCOOH、AcOH和TFA这3种酸性添加剂对洗脱效果的影响。结果如图3c所示，添加了酸的洗脱液中L-PeakⅡ成分峰面积均较50% ACN水溶液更大，其中添加了0.2% TFA的洗脱液中L-PeakⅡ峰面积最大，且峰形对称，表明该洗脱溶剂对L-PeakⅡ成分具有最佳的洗脱效果。故研究选择含0.2% TFA的50% ACN水溶液，作为富集的洗脱溶剂。

#### 2.3.4 洗脱时间优化

此外，对洗脱时间也进行了优化。在洗脱过程中，材料吸附的多肽解吸后，如果继续洗脱则会促进材料再一次对多肽进行吸附。本实验考察了不同洗脱时间（1、2、4、10、16 h）时L-PeakⅡ的洗脱情况。实验结果如图3d所示，随着洗脱时间的增加，多肽L-PeakⅡ的峰面积呈先增加后减少的变化趋势，多肽解吸量在2 h达到最大值，而后随着洗脱时间的增加，材料与多肽再次吸附导致洗脱下的多肽量减少，故在2 h时富集效果最好。因此选择2 h作为最佳洗脱时间。

### 2.4 富集方法重复性考察

为了考察PCN-222（Fe）富集地龙多肽的方法重复性，在最佳吸附和洗脱条件下，平行进行6份材料富集实验，使用同一份酶解液，然后利用HPLC分析。统计得6份洗脱液中L-PeakⅡ色谱峰峰面积的RSD为2.85%。表明研究所建立的PCN-222（Fe）材料富集地龙多肽L-PeakⅡ方法具有可重复性。

### 2.5 L-PeakⅡ制备

使用制备型液相色谱仪制备多肽L-PeakⅡ，并在1.6节条件下对制备组分进行检测。分析结果如图4所示，制备组分的出峰时间在15 min左右，与酶解液的HPLC图谱的目标色谱峰一致，表明收集的组分确为L-PeakⅡ。制备后的L-PeakⅡ组分相较于酶解液的色谱图更为纯净，为单一色谱峰，峰形对称，峰面积百分比达到95%以上，纯度较高。综上表明，通过PCN-222（Fe）材料富集结合制备色谱技术，可以得到高纯度地龙多肽L-PeakⅡ。相较于传统多肽分离纯化方法，研究所建立的方法具有简便易行、速度快、效率高的优势。

**图4:**
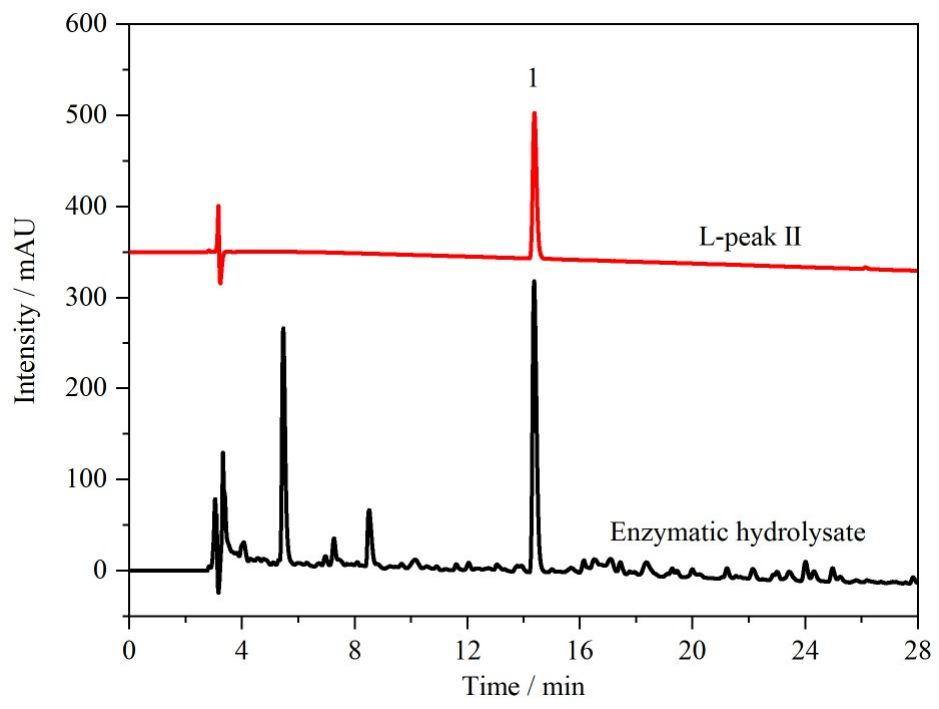
L-PeakⅡ制备组分与酶解溶液的HPLC对比图

## 3 结论

本文采用PCN-222（Fe）材料与反相液相色谱技术联合，使用金属有机框架材料PCN-222（Fe）作为吸附剂，实现对地龙多肽L-PeakⅡ快速高效的分离纯化，并通过制备型液相色谱制备得到高纯度多肽L-PeakⅡ组分，实现了地龙药材样品中多肽成分的高效分离提取，拓展了PCN-222（Fe）材料在传统中药材上的应用，为今后进一步探索地龙中多肽成分活性L-PeakⅡ的研究提供了快速提取分离的技术支持，对其质量控制、实际生产及临床应用具有重要的意义，且有助于推动虫类中药活性肽类物质的基础研究与质量评价。
